# IPS-1 differentially induces *TRAIL*, *BCL2*, *BIRC3* and *PRKCE* in type I interferons-dependent and -independent anticancer activity

**DOI:** 10.1038/cddis.2015.122

**Published:** 2015-05-07

**Authors:** S Kumar, H Ingle, S Mishra, R S Mahla, A Kumar, T Kawai, S Akira, A Takaoka, A A Raut, H Kumar

**Affiliations:** 1 Laboratory of Immunology, Department of Biological Sciences, Indian Institute of Science Education and Research, Bhopal, India; 2 Laboratory of Molecular Immunobiology, Graduate School of Biological Sciences, Nara Institute of Science and Technology, Nara, Japan; 3 Laboratory of Host Defense, WPI Immunology Frontier Research Centre, Osaka University, Osaka, Japan; 4 Division of Signaling in Cancer and Immunology, Institute for Genetic Medicine, Hokkaido University, Sapporo, Japan; 5 Pathogenomics Laboratory, ICAR-National Institute of High Security Animal Diseases, OIE Reference lab for Avian Influenza, Bhopal, India

## Abstract

RIG-I-like receptors are the key cytosolic sensors for RNA viruses and induce the production of type I interferons (IFN) and pro-inflammatory cytokines through a sole adaptor IFN-*β* promoter stimulator-1 (IPS-1) (also known as Cardif, MAVS and VISA) in antiviral innate immunity. These sensors also have a pivotal role in anticancer activity through induction of apoptosis. However, the mechanism for their anticancer activity is poorly understood. Here, we show that anticancer vaccine adjuvant, PolyIC (primarily sensed by MDA5) and the oncolytic virus, Newcastle disease virus (NDV) (sensed by RIG-I), induce anticancer activity. The ectopic expression of IPS-1 into type I IFN-responsive and non-responsive cancer cells induces anticancer activity. PolyIC transfection and NDV infection upregulate pro-apoptotic gene *TRAIL* and downregulate the anti-apoptotic genes *BCL2*, *BIRC3* and *PRKCE*. Furthermore, stable knockdown of IPS-1, IRF3 or IRF7 in IFN-non-responsive cancer cells show reduced anticancer activity by suppressing apoptosis via *TRAIL* and anti-apoptotic genes. Collectively, our study shows that IPS-1 induces anticancer activity through upregulation of pro-apoptotic gene *TRAIL* and downregulation of the anti-apoptotic genes *BCL2*, *BIRC3* and *PRKCE* via IRF3 and IRF7 in type I IFN-dependent and -independent manners.

The primary protection of the host from various pathogens is ensured by the innate immune system, which consists of families of sensors such as the Toll-like receptors (TLRs), RIG-I-like receptors (RLRs) and NOD-like receptors. These sensors recognize the diverse range of pathogens in various cellular compartments and lead to the activation of innate immunity, including the production of various cytokines that create an anti-pathogenic environment to limit the pathogen. RLRs are cytosolic sensors that recognize the viral RNA and recruit an adaptor, Interferon (IFN)-*β* promoter stimulator-1 (IPS-1), also known as CARDIF, MAVS or VISA. IPS-1, a protein that contains a caspase activation and -recruitment domain (CARD), is localized to the mitochondria for its antiviral function.^1, 2, 3, 4^ Mice lacking IPS-1 show severely impaired antiviral innate immunity.^5^ The RLRs/IPS-1 signaling axis activates a cascade of signals that predominantly induces the production of the type I IFN and pro-inflammatory cytokines through IRFs and NF-*κ*B, respectively, to establish an antiviral state.

In addition to the pivotal role that host immunity has against numerous pathogen challenges, it is crucial in immune surveillance against altered-self cells. Immune mediators such as cytokines, chemokines and type I IFN initiate a complex network of signals to induce an anti-tumor state by triggering various biochemical processes such as cell cycle arrest and apoptosis. Additionally, these immune mediators facilitate cytotoxicity to the tumor cells through the recruitment of immunocompetent cells. The cytotoxic activity is mediated through the upregulation of pro-apoptotic genes and the downregulation of anti-apoptotic genes. These changes are critical for cancer cell death.^6^ Various innate and adaptive cytokines are used for treatment of several types of cancer.^7, 8^ The type I IFN are essential for antiviral immunity and induce pleiotropic effects such as the inhibition of malignant growth and apoptosis of altered-self cells.

In addition, pathogen-associated molecular patterns such as polyinosinic:polycytidylic acid (polyIC), a synthetic analog of double-stranded RNA and viruses known as oncolytic viruses such as Vesicular stomatitis virus, Newcastle disease virus (NDV) and Sendai virus induce anticancer activity.^9^ However, the molecular mechanisms for these agents are poorly understood.

Here, we showed that treatment of cancer cells with polyIC transfection or NDV infection initiates RIG-I- and MDA5-dependent anticancer activity through recruitment of an adaptor, IPS-1. Using IFN *α*/*β* receptor1 (*IFNAR*1)-sufficient and *IFNAR*1-deficient cancer cells, we showed that these anticancer activities require the RLR signaling pathway. However, type I IFN are dispensable for the anticancer activity. The RLR pathway induces anticancer activity through the selective induction of cell death or apoptosis via upregulation of the pro-apoptotic gene *TRAIL* and downregulation of the anti-apoptotic genes *BCL2*, *BIRC3* and *PRKCE.* These changes lead to post-translational activation of caspases −3 and −9 and PARP-1 in cancer cells. Furthermore, our study reveals that IFN regulatory factors (IRF)3 and IRF7 are indispensable for the RLR-mediated anticancer activity.

## Results

### PolyIC and NDV induces anticancer activity

In cancer therapy, polyIC is used as an adjuvant.^10^ However, the mechanism for the polyIC-induced anticancer activity is poorly understood. *In vitro* or *in vivo* polyIC challenge induces a TLR3- and MDA5-dependent signaling pathway.

**Figure 1:**
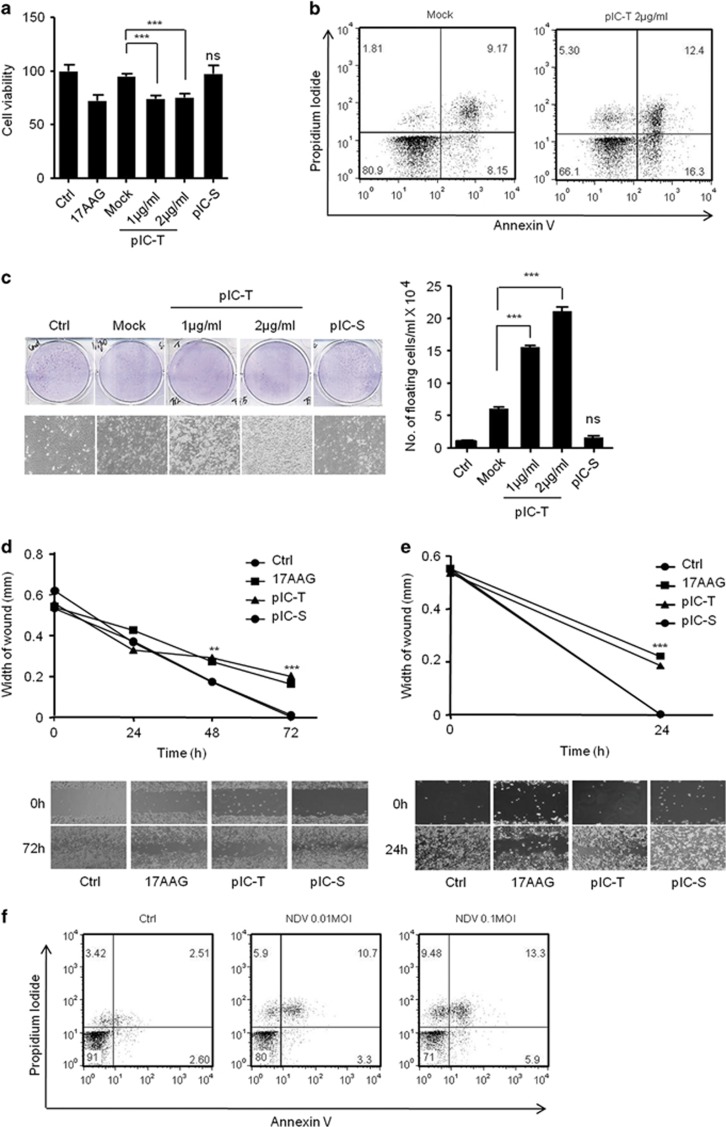
PolyIC transfection and NDV infection induce anticancer activity in various cancer cells. (**a)** HEK293T **(b)** MDAMB-231 cells were transfected with the indicated concentrations of polyIC (pIC-T) or stimulated with 25 *μ*g/ml of polyIC (pIC-S) along with the appropriate controls. After 24 h, the cell viability was determined using the MTT assay **(a)** or the cells were stained with API, and the level of apoptosis was determined using flow cytometry **(b),** 17AAG is a anticancer drug, used as a positive control. **(c)** Clonogenic assay: colonies of MDAMB-231 cells were pIC-T with the indicated concentrations or pIC-S with 25 *μ*g/ml, and the numbers of colonies in the plates were examined after 48 h. The left panel shows the microscopic images, and the right panel shows the numbers of dead cells or floating cells after different treatments. **(d)** IMR32 and **(e)** MDAMB-231 cell monolayers were pIC-T at 2 *μ*g/ml or pIC-S with 25 *μ*g/ml and wounds were scratched at 0 h. The upper panel shows the wound healing measured at 24, 48 and 72 h **(d)** or only at 24 h (**e**). The lower panel shows the wounds observed using light microscopy at 72 h **(d)** and 24 h **(e)**. **(f)** MDAMB-231 cells were infected with the indicated MOI of NDV. After 24 h, the cells were stained with annexin V/PI, and apoptosis was determined by using flow cytometry

To investigate the role of the polyIC-induced anticancer activities, including anti-proliferation and apoptosis. The HEK293T cells were transfected or stimulated with polyIC, transfection in HEK293T cells showed marked reduction in cell viability compared to polyIC-stimulation and control cells evaluated using the MTT assay, similar to that of 17AAG-treated cells (Figure 1a). 17AAG (17- *N*-allylamino-17-demethoxygeldanamycin) is an anticancer drug and was used as positive control. Furthermore, we tested the ability of polyIC to induce apoptosis using flow cytometric analysis of annexin V/propidium iodide (API) staining using MDAMB-231 breast cancer cells. The polyIC-transfected MDAMB-231 cells showed increases in API-positive apoptotic cells compared with control (Figure 1b). Next, we confirmed the anticancer activity by polyIC transfection or -stimulation by using the colony-formation assay and wound healing assay (WHA) to evaluate the proliferation and migration properties of the cancer cells. PolyIC transfection caused a marked reduction in number of colonies and a delayed wound healing in the neuronal cancer cells IMR32, and MDAMB-231 cells (Figures 1c–e). These observation prompted us to investigate whether these cells expresses TLR3 and induce TLR3-dependent signaling. To this end, we first tested the expression of TLR3 in HEK293T, MDAMB-231 and IMR32 cells using semi-quantitative PCR and quantitative real-time PCR (qRT-PCR) (Supplementary Figure S1).We found that HEK293T cells expresses low level of TLR3 compared to other tested cell lines. Next, we transfected or stimulated these cells with polyIC and quantified the mRNA levels of *IFNβ* and *IL-6*. HEK293T and IMR32 induced *IFNβ* after polyIC-stimulation. On other hand, MDAMB-231 cells induced *IL-*6 but not *IFNβ* (Supplementary Figure S1). Collectively, our results suggest that PolyIC stimulation induce low level of cytotoxic effects compared with PolyI:C-transfection suggesting RLR signaling pathway is having a predominant role in the induction of cytotoxic effect compared with TLR3 signaling pathway.

To understand the role of the RIG-I-dependent pathway in the anticancer activity, we used NDV, which is sensed by RIG-I, and tested whether it induces anticancer effects. MDAMB-231 cells were infected with NDV for 24 h and analyzed using API. At multiplicity of infection (MOI)s of 0.01 and 0.1, the infected cells showed dose-dependent apoptosis compared with the control (Figure 1f). Collectively, our results suggest that the RLR-dependent pathway is crucial in the anticancer activity.

### IPS-1 induces anticancer activity in various cancer cell lines

**Figure 2:**
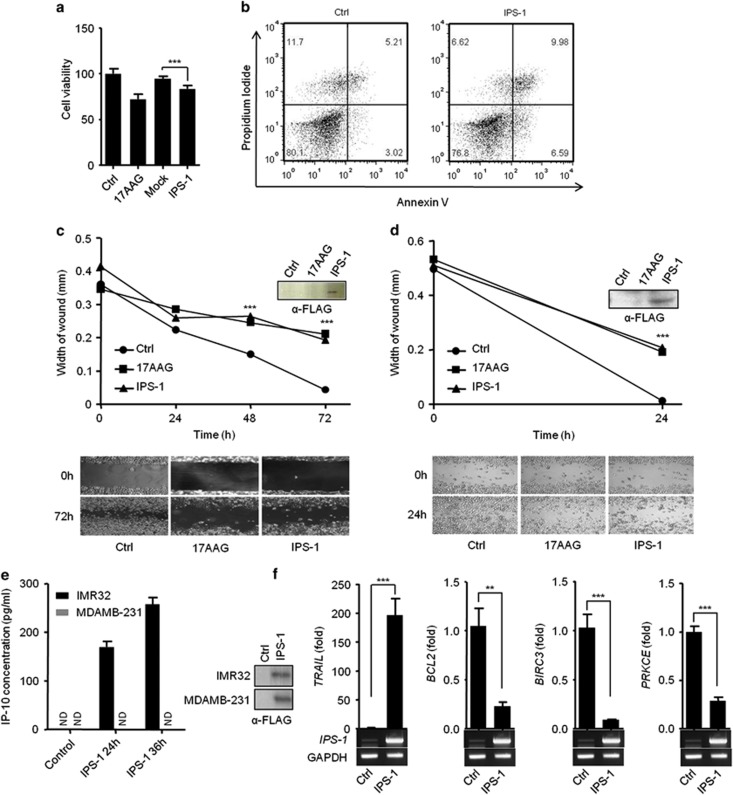
IPS-1 induces anticancer properties in various cancer cell lines. **(a)** HEK293T or **(b)** MDAMB-231 cells were transiently transfected with IPS-1, and 24 h later, the cell viability was determined using the MTT assay **(a)** or the cells were stained with annexin V/PI, and apoptosis was determined using flow cytometry **(b)**. **(c)** IMR32 and **(d)** MDAMB-231 cell monolayers were transiently transfected with a FLAG-IPS-1 expression plasmid, and wounds were scratched at 0 h. The upper panel shows the wound healing measured at 24, 48 and 72 h (**c**) or only at 24 h (**d**). The bottom panel shows the wounds observed using light microscopy at 72 h (**c**) and 24 h (**d**). At the end of the experiment, the cell lysates were analyzed for the expression of IPS-1 by immunoblot analysis using an anti-FLAG-IPS-1 antibody (right). **(e)** FLAG-IPS-1 was transiently transfected in IMR32 and MDAMB-231 cells and cells supernatants were collected at indicated time-points to measure the level of IP-10 cytokine, and IPS-1 over-expression were confirmed by western blot using anti-FLAG antibody (right panel). **(f)** HEK293T cells were transiently transfected with the FLAG-IPS-1 plasmid. After 24 h, the cells were lysed to quantify the mRNA levels of apoptotic genes using qRT-PCR, and the over-expression of IPS-1 was confirmed by semi-quantitative PCR (below)

PolyIC transfection activates RLR sensors to induce signaling through the recruitment of an adaptor, IPS-1. Therefore, our previous results prompted us to investigate the involvement of IPS-1 in the anticancer activity. The ectopic expression of IPS-1 in HEK293T cells resulted in a significant reduction in the cell viability (Figure 2a). Additionally, IPS-1 over-expression in the MDAMB-231 cells resulted in apoptosis, which was further confirmed by using the API assay (Figure 2b). Finally, transient expression of IPS-1 in IMR32 and MDAMB-231 cells resulted in a significant reduction in the wound healing compared with the control cells, suggesting that IPS-1 may have a crucial role in the anticancer activity (Figures 2c and d). To test the type I IFN responses, we transiently transfected IPS-1 in IMR32 and MDAMB-231 cells and measured the levels of type I IFN-inducible cytokine IP-10 in culture supernatant by ELISA. Surprisingly, IMR32 cells are type I IFN sufficient, on other hand, MDAMB-231 cells are type I IFN deficient (Figure 2e). To understand the molecular mechanism of the IPS-1-induced apoptosis, we used qRT-PCR to screen for changes in the mRNA expression levels of various cancer-associated genes following IPS-1 transfection. Interestingly, the pro-apoptotic gene, tumor necrosis factor-related apoptosis-inducing ligand (*TRAIL*) was upregulated. In contrast, the expression levels of the anti-apoptotic genes B-cell lymphoma2 (*BCL2*), Baculoviral IAP repeat-containing protein3 (*BIRC3*) and protein kinase C epsilon (*PRKCE*) were downregulated significantly compared to control (Figure 2f). The expression levels of several other studied genes remained unchanged (Supplementary Table S1).

### Knockdown of IPS-1 reduces anticancer activity

**Figure 3:**
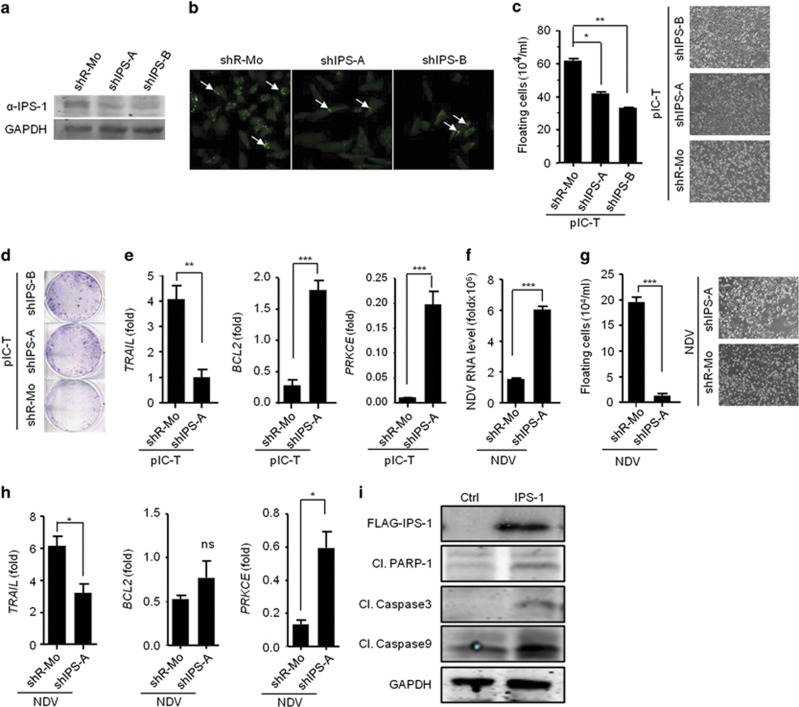
IPS-1 is required for anticancer activity. The efficacy of shRNA-mediated stable knockdown of IPS-1 in the MDAMB-231 cells (shIPS-A and -B) was validated by quantifying the protein levels of IPS-1 in the cells by **(a)** immunoblot and **(b)** confocal microscopic analysis using an anti-IPS-1 antibody. **(c)** shIPS-A and -B cells were transfected with polyIC, and 24 h later, the numbers of floating cells (dead cells) were counted (left) and images were taken (right). **(d)** shIPS-A and -B cells were grown until they formed colonies, which were transfected with polyIC. After 2 days, the numbers of colonies in the plates were examined using the clonogenic assay. **(e)** shIPS-A cells were transfected with polyIC. After 6 h, the mRNA levels of the indicated genes were quantified using qRT-PCR. The shIPS cells were infected with MOI=5 of NDV, and 24 h later, the mRNA levels of the NDV transcripts **(f)** and floating cells were counted and microscopic images were taken **(g)**, or other genes as indicated were quantified using qRT-PCR **(h)**. **(i)** MDAMB-231 cells were transiently transfected with a FLAG-IPS-1 expression plasmid. After 24 h, the cells were lysed, and the protein levels of the indicated genes were quantified by immunoblot (Cl., Cleaved)

To know whether IPS-1 is dispensable or not, we employed shRNA-mediated knockdown strategy to reduce the endogenous expression of IPS-1 in MDAMB-231 cells. MDAMB-231 cells expressing one of two distinct shRNA, shIPS-1-A or shIPS-1-B, were generated, and these cells showed a marked reduction in endogenous IPS-1 compared with the non-specific shRNA-transfected cells, as shown by immunoblot and confocal microscopy using an anti-IPS-1 antibody (Figures 3a and b). We next analyzed the cancerous properties in these cells after polyIC transfection. We found significantly lower levels of cell death in the IPS-1-targeted shRNA cells (shIPS-A and shIPS-B) compared with the control shRNA-treated cells (shR-Mo) (Figure 3c). Notably, polyIC transfection induced anticancer activity via cell death and resulted in an increase in the number of floating cells. We further performed colony-formation assay after polyIC transfection and consistent with the previous result, the anticancer activity was reduced in the shIPS-1 cells, as shown by a significant increase in the number of colonies (Figure 3d). These results indicated that IPS-1 is necessary to promote anticancer activity. Next, we examined the expression levels of *TRAIL*, *BCL2* and *PRKCE* upon polyIC transfection. We found that *TRAIL* expression was significantly reduced in the shIPS-1 cells, whereas the expression of *BCL2* and *PRKCE* was significantly increased (Figure 3e).

We also determined the role of IPS-1 in the NDV-mediated cancer cell death by infecting shIPS-1 cells with NDV. Although shIPS-1 cells showed enhanced viral replication,we observed a significant decrease in the NDV-mediated cell death (Figures 3f and g). Furthermore, the shIPS-1 cells showed a significant decrease in the levels of *TRAIL* expression and in contrast, *BCL2* and *PRKCE* were modestly upregulated (Figure 3h), but the expression of *BIRC3* remained unchanged (Supplementary Figure S2). Finally to test caspase activation, we over-expressed IPS-1 in MDAMB-231 cells and analyzed the caspase-3, -9 and PARP-1 cleavage by immunoblot using specific antibodies. The results indicated that IPS-1 induced the cleavage of caspase−3, −9 and PARP-1 (Figure 3i). These data collectively demonstrated that IPS-1 is pivotal in inducing anticancer activity by upregulating the pro-apoptotic gene *TRAIL* and downregulating anti-apoptotic genes such as *BCL2* and *PRKCE* in caspase-dependent manner.

### IPS-1 induces type I IFN-dependent anticancer activity

**Figure 4:**
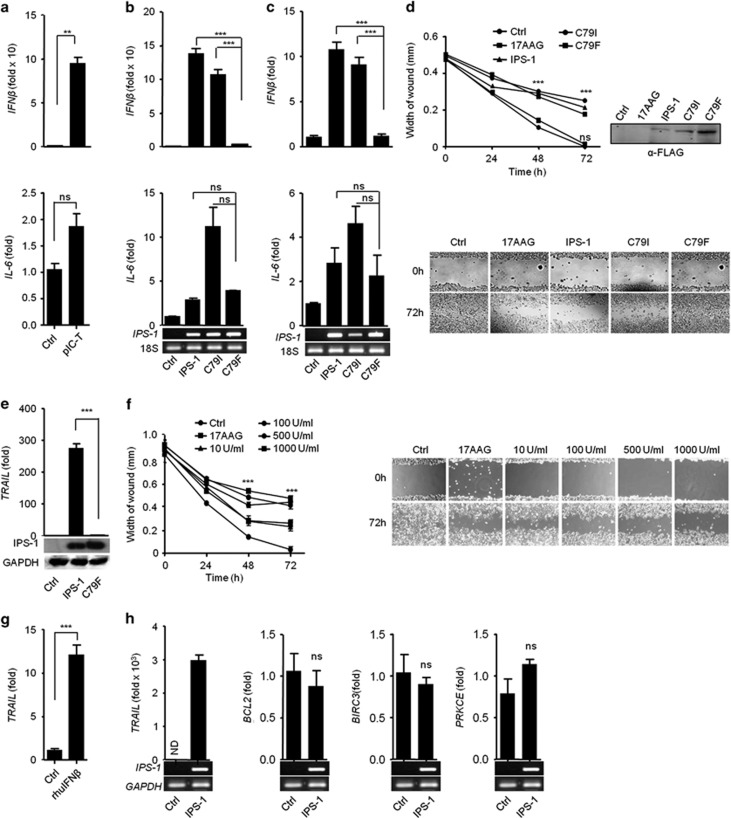
PolyIC transfection and IPS-1 over-expression induce the production of type I IFN, demonstrating anticancer activity. **(a)** IMR32 cells were transfected with polyIC, and 24 h later, the cells were lysed to quantify the mRNA level of various cytokines by qRT-PCR. **(b)** HEK293T and **(c)** IMR32 cells were transfected with IPS-1, C79I or C79F plasmids, and 24 h later, the levels of various cytokines quantified by qRT-PCR. IMR32 cell monolayers were transfected with IPS-1, C79I or C79F plasmids **(d)** or stimulated with indicated concentrations of recombinant IFN*β* **(f)**, and wounds were scratched at 0 h. The wound healing was measured at 24 h time intervals and observed using light microscopy. HEK293T cells were transfected with either IPS-1 or C79F **(e)** IMR32 cells were treated with rhuIFN*β* **(g)** or transfected with IPS-1 plasmid **(h)**. The mRNA level of *TRAIL* was quantified by qRT-PCR. Finally, the over-expression of IPS-1 or C79F was confirmed by semi-quantitative PCR or immunoblot analysis (lower panels)

The RLRs pathway induces IPS-1-dependent type I IFN and pro-inflammatory cytokines. Therefore, we further investigated whether RLR/IPS-1-dependent responses are involved in the anticancer activity. To this end, IMR32 cells were transfected with polyIC, and found the expression of *IFNβ* was strongly induced, whereas *IL-6* was marginally induced compared with control by qRT-PCR (Figure 4a).

To investigate the roles of IPS-1-induced type I IFN in anticancer activity, several mutations were created in the CARD of IPS-1 and screened to find mutant(s) that abrogated type I IFN but not pro-inflammatory cytokines. First, we identified conserved amino acids by comparing the IPS-1-CARD from different species using CLUSTAL W (Supplementary Figure S3). We used site-directed mutagenesis to construct non-synonymous mutants of IPS-1 with the following changes: R43W, R64Q, R65W, G67S, C79I and C79F. These constructs were transfected into HEK293T cells, and the expression was verified by immunoblot (Supplementary Figure S4). Next, the mutants were tested for the ability to activate the IFN*β* and NF-*κ*B promoters by using the luciferase assay. Mutants R64Q and R65W did not activate these targets, but the R43W and G67S mutants reduced both the IFN*β* and NF-*κ*B promoter activities compared with the wild-type IPS-1 (WT-IPS-1) (Supplementary Figure S5). Mutant C79I induced IFN*β* and NF-*κ*B promoter activities comparable to wild-type (WT)-IPS-1 (Supplementary Figure S5). In contrast, C79F did not activate the IFN*β* promoter, whereas NF-*κ*B activity was reduced compared with WT and C79I (Supplementary Figure S5). However the interaction of C79I and C79F with CARD of MDA5 was remaining unchanged (Supplementary Figure S6).

The C79I or C79F mutants were further validated by qRT-PCR for expression of *IFNβ* and *IL-6* in IMR32 and HEK293T cells. WT-IPS-1 and C79I induced comparable levels of *IFNβ* in IMR32 cells and HEK293T cells, whereas C79F failed to induce *IFNβ*. Additionally, *IL-6* expression was not significantly different among the WT, C79I and C79F mutants in HEK293T (Figure 4b) and IMR32 cells (Figure 4c). We further used WT-IPS-1, C79I and C79F to understand the roles of type I IFN and pro-inflammatory cytokines in the anticancer activity. IMR32 cells were transfected with WT-IPS-1, C79I or C79F mutants and subjected to the WHA. Both WT-IPS-1 and C79I significantly inhibited migration toward the wound, whereas C79F caused no inhibition in the migration, similar to control (Figure 4d). These results suggest that the type I IFN induced by WT and C79I may possess anticancer activity. To confirm the involvement of IFN*β* in the anticancer activity, IMR32 cells were treated with recombinant human IFN*β* (rhuIFN*β*), and the anticancer activity was evaluated using the WHA. The rhuIFN*β* inhibited the migration of the cells in a dose-dependent manner (Figure 4f). Furthermore, we treated IMR32 cells with IFNAR1 blocking antibody before polyIC transfection and measured the cytotoxicity by cell viability assay. We found that blocking of IFNAR1 resulted in decrease of cytotoxicity compared with polyIC transfection alone (Supplementary Figure S7), suggesting that type I IFNs is crucial for anticancer activity.

To investigate the underlying molecular mechanism for the IFN*β*-based anticancer activity, we treated IMR32 cells with rhuIFN*β* and found an upregulation of *TRAIL* (Figure 4g). Furthermore, IPS-1 over-expression in IMR32 cells resulted in the strong upregulation of *TRAIL,* but the levels of other anti-apoptotic genes remained unchanged (Figure 4h), indicating that the IPS-1-dependent type I IFN play a crucial role in anticancer activity through the upregulation of the pro-apoptotic gene *TRAIL*. Finally, the ability of C79F to induce *TRAIL* was tested in the HEK293T cells. IPS-1 strongly induced *TRAIL,* but C79F over-expression failed to induce *TRAIL* (Figure 4e). Taken together, these data suggest that IPS-1 induces the type I IFN, which in turn induce the pro-apoptotic gene *TRAIL*, resulting in anticancer activity.

### IPS-1 exhibits type I IFN-independent anticancer activity

**Figure 5:**
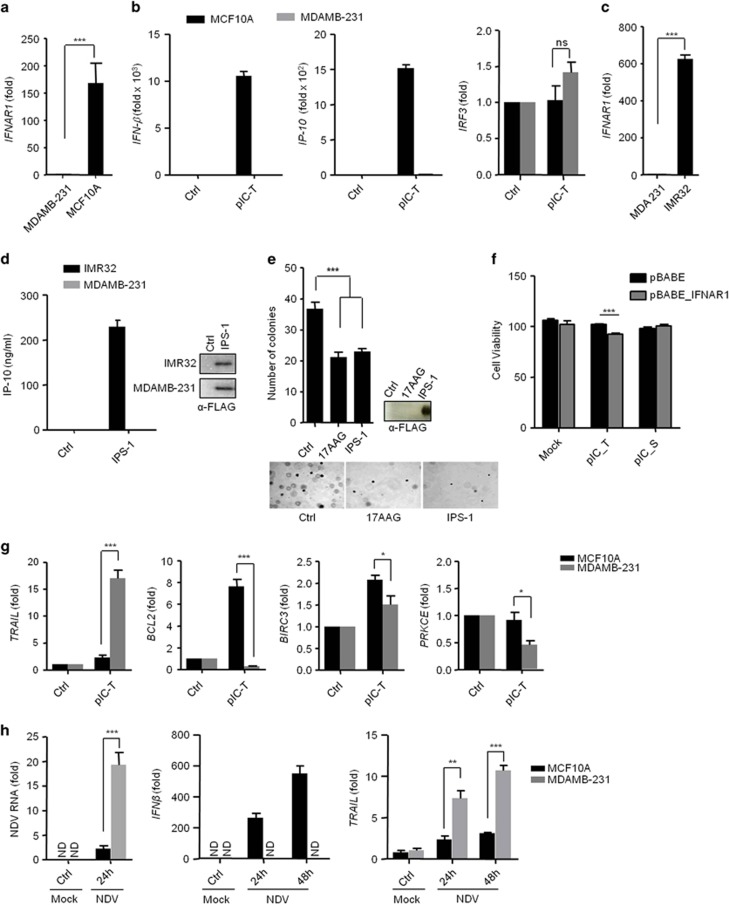
IPS-1 induces type I IFN-independent anticancer activity. **(a)** MDAMB-231 and MCF10A cells were lysed and the expression levels of *IFNAR*1 were quantified by qRT-PCR. **(b)** MCF10A and MDAMB-231 cells were transfected with polyIC. After three hours cells were lysed, and the mRNA levels of IFN*β*, IP-10 and IRF3 were quantified by qRT-PCR. **(c)** MDAMB-231 and IMR32 cells were lysed and the expression levels of *IFNAR*1 were quantified by qRT-PCR. **(d)** MDAMB-231 and IMR32 cells were transfected with IPS-1 plasmid. After 36 h, the supernatants were collected, and the levels of the IP-10 cytokine were measured by ELISA. The over-expression of IPS-1 was confirmed by immunoblot analysis. **(e)** MDAMB-231 cells transiently transfected with FLAG-IPS-1 were subjected to the anchorage-independent colony-formation assay. (**e**) All cells were treated with the anticancer drug 17AAG (used as a positive control). At the end of experiment, the cell lysates were analyzed for the expression of IPS-1 by immunoblot analysis using an anti-FLAG-IPS-1 antibody (right). **(f)** MDAMB-231 cells were infected with pBABE or pBABE-IFNAR1 retrovirus and maintained for 48 h. These cells were seeded into 96-well plate followed by polyIC transfection or stimulation, after 30 h, cells viability assay was performed. MDAMB-231 and MCF10A cells were transfected with polyIC for 3 h **(g)** or NDV infection for 24 h **(h).** The cells were lysed and the mRNA levels of the indicated genes were quantified by using qRT-PCR

IPS-1 induces anticancer properties in MDAMB-231 breast cancer cells, and it has previously been shown that MDAMB-231 cells have compromised IFNAR1 expression.^11, 12^ *IFNAR1* is required for IFN production in autocrine and paracrine manners. Therefore, we first evaluated the IFNAR1 expression in MDAMB-231 cells and the normal breast cell line, MCF10A, by qRT-PCR. The MDAMB-231 cells did not show expression of *IFNAR*1 compared with MCF10A cells (Figure 5a). Further, we quantified the mRNA levels of *IFNβ* and the IFN-inducible gene, *IP-10,* in MDAMB-231 and MCF10A cells after polyIC transfection. As expected, the *IFNAR*1-sufficient MCF10A cells produced large amounts (~10,000- and 1500-fold increases) of *IFNβ* and *IP-10*, respectively, compared to the *IFNAR*1-deficient MDAMB-231 cells, however, *IRF3* expression was comparable (Figure 5b). Then, we compared IFNAR1 expression in the IMR32 and MDAMB-321 cells. The IMR32 cells showed elevated expression of *IFNAR*1 compared to the MDAMB-231 cells (Figure 5c). Furthermore, IMR32 and MDAMB-231 cells were transfected with the IPS-1 plasmid, and 24 h later, the culture supernatants were tested for IP-10 production by ELISA. The supernatants from the MDAMB-231 cells did not show IP-10 production compared with the IMR32 cells (Figure 5d), suggesting that MDAMB-231 cells lacked IFNAR1 expression and did not promote the type I IFN-dependent responses. Further, we determined the anchorage-independent growth in cancer cells by using the soft agar assay after IPS-1 over-expression. Consistent with the previous results, MDAMB-231 cells expressing IPS-1 show elevated anticancer activity (Figure 5e). To understand further the role of type I IFN, we have complemented IFNAR1 in MDAMB-231 cells using retroviral system and tested the cytotoxic effects after polyIC transfection. We found that complementation of IFNAR1 increased the cytotoxicity, suggesting type I IFNs also have an important role in cytotoxicity (Figure 5f). To understand the underlying mechanism of the IPS-1-dependent *IFNβ*-independent anticancer activity, we transfected MCF10A and MDAMB-231 cells with polyIC, and the expression of *TRAIL*, *BCL2*, *BIRC3* and *PRKCE* was tested by qRT-PCR (Figure 5g). We found the selective upregulation of *TRAIL* and downregulation of *BCL2*, *BIRC3* and *PRKCE* in the MDAMB-231 cancer cells but not in the MCF10A cells. Together, these results suggest that IPS-1 induces anticancer activity in an *IFNβ*-independent manner by regulating *TRAIL*, *BCL2*, *BIRC3* and *PRKCE*. Previous reports have suggested that NDV replicates selectively in various cancer cells due to an impaired ability to produce type I IFN. We therefore infected MCF10A and MDAMB-231 cells with NDV (MOI=5) and measured the viral load, *IFNβ* and *TRAIL* mRNA level at 12 h and 24 h by qRT-PCR (Figure 5h). The hemagglutinin-neuraminidase (HN) protein of NDV has been reported to induce the expression of TRAIL and type I IFNs.^13^ MCF10A cells showed less NDV burden due to the ability to produce more type I IFN. However, in MDAMB-231 cells viral load and HN protein is higher due to lack of type I IFNs and resulted to induction of higher levels of *TRAIL* compared with MCF10A.

### IPS-1-induces *TRAIL* in IRF3- and IRF7-dependent manners to induce apoptosis in cancer cells

**Figure 6:**
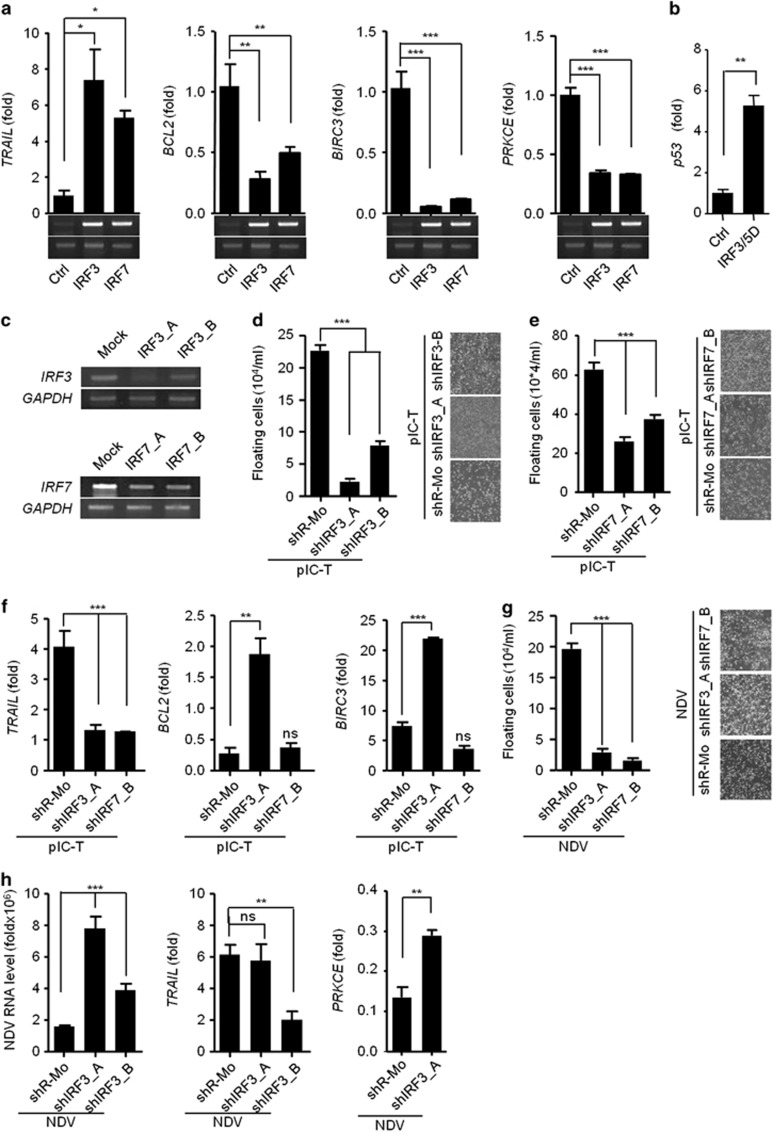
IPS-1 induces IRF3- and IRF7-dependent *TRAIL* to induce apoptosis in MDAMB-231 cells **(a)** HEK293T cells were transiently transfected with IRF3 or IRF7 expression plasmids, and 24 h later, the cells were lysed to quantify the mRNA levels of apoptotic genes by qRT-PCR. The over-expression of IRF3 and IRF7 was confirmed by semi-quantitative PCR (below). **(b)** MDAMB-231 cells were transiently transfected with IRF3/5D, after 24 h, cells were lysed and the mRNA level of p53 was quantified using qRT-PCR. **(c)** The MDAMB-231 cells were knocked down for IRF3 (shIRF3) or IRF7 (shIRF7) by specific shRNA and expression of IRF3 and IRF7 were analysed by semi-quantitative PCR, using GAPDH as the loading control. **(d)** Cells stably expressing shIRF3 or **(e)** shIRF7 cells were transfected with polyIC, and 24 h later, the numbers of floating cells were counted (left) and imaged by microscopy (right). **(f)** Cells stably expressing shIRF3 and shIRF7 cells were transfected with polyIC, and 3 h later, the mRNA levels of the indicated genes were measured by qRT-PCR. **(g)** shIRF3 cells were infected with NDV at an MOI of 5, and 24 h later, the numbers of floating cells were counted (left) and imaged by microscopy (right), and **(h)** the NDV transcripts, *TRAIL*, *BCL2* and *PRKCE* mRNA levels were quantified by qRT-PCR

The RLR-IPS-1 signaling axis is known to activate the IRF3 and IRF7 transcription factors. We therefore examined the role of IRF3 and IRF7 in IPS-1-mediated anticancer activity through induction of *TRAIL*. To this end, HEK293T cells were transfected with IRF3 and IRF7 expression plasmids. IRF3 and IRF7 both induced similar levels of *TRAIL* and diminished the levels of *BCL2*, *BIRC3* and *PRKCE* (Figure 6a). To understand the underlying mechanism for downregulation of these genes, we over-expressed the active form of IRF3 (IRF3/5D) in MDAMD-231 cells^14^ and measured the mRNA levels of various transcription factors such as SETB1, DDIT3 and p53 which was known to bind to the promoter of BCL2 for its downregulation.^15, 16, 17^ We found that p53 was upregulated upon over-expression of IRF3/5D and therefore suppresses anti-apopototic gene (Figure 6b). Moreover, stable knockdown in MDAMB-231 cells of IRF3 was achieved using shRNA for IRF3 or shIRF3 (shIRF3_A and shIRF3_B), and knockdown of IRF7 was similarly achieved using shRNA for IRF7 (shIRF7_A and shIRF7_B) (Figure 6c). Using these cells, we investigated the role of IRF3 and IRF7 on the anticancer activity after polyIC transfection. The shIRF3 and shIRF7 cells were found to be resistant to cell death because the numbers of floating cells were significantly reduced (Figures 6d and e). Further experiments were performed using the most effectively knocked down clones of IRF3 (shIRF3_A) or IRF7 (shIRF7_B). We analyzed the expression of *TRAIL*, *BCL2* and *BIRC3* in shIRF3 and shIRF7 cells after polyIC transfection and found that the expression of *TRAIL* was reduced in both the cells. shIRF3 cells showed significant increases in the levels of *BCL2* and *BIRC3* compared with shR-Mo-treated cells (Figure 6f). The shIRF7 cells did not induce *BCL2* and *BIRC3* (Figure 6f). The expression of the *PRKCE* gene was comparable in both shIRF3- and shIRF7-treated cells and was similar to the control cells (Supplementary Figure S8).

Next, we examined anticancer activity in both shIRF3 clones after NDV infection. Similar to the responses to polyIC, the shIRF3 cells showed significant less cell death after NDV infection (Figure 6g), and the cells were more permissive to NDV replication (Figure 6h). We also investigated the expression levels of pro- and anti-apoptotic genes. The shIRF3_A cells showed a significant reduction in the levels of *TRAIL,* whereas *PRKCE* was modestly upregulated (Figure 6i). The expression of *BCL2* and *BIRC3* was not changed (Supplementary Figure S9). These results demonstrate that RLR-IPS-1 signaling through IRF3 and IRF7 induces anticancer activity by modulating the expression of pro-apoptotic and anti-apoptotic genes.

## Discussion

RIG-I and MDA5-dependent signaling activates IPS-1 to induce antiviral immunity through the induction of type I IFN, inflammatory cytokines and apoptosis.^18, 19, 20, 21, 22, 23^ This pathway also has a crucial role in the induction of apoptosis in cancer cells.^12, 24, 25^ PolyIC and NDV are sensed by RLR sensors and have been used in cancer therapy.^26, 27, 28^ Previously, it has been reported that polyIC induces apoptosis in cancer cells *via* the TLR3 and RLR pathway.^29, 30, 31, 32^ We found that activation of RLRs by polyIC and NDV in IMR32 and MDAMB-231 is predominant in inducing cytotoxic activity. We also found that the ectopic expression of IPS-1 in these cancer cells promotes apoptosis and hence reduces the cellular viability. Additionally, IPS-1 induces anti-migratory effects and delays wound healing in IMR32 and MDAMB-231 cells, as shown by the WHA. In contrast, the knockdown of endogenous IPS-1 in cancer cells significantly reduced apoptosis, resulting in a reduction in the anticancer activity after polyIC transfection or NDV infection. Thus, both the over-expression and knockdown data indicate the pivotal role of IPS-1 in anticancer activity.

Previous reports show that activation of RLR induces TRAIL, Noxa and Puma, but the expression of these genes was independent of the cancerous properties.^12^ In our experiment, we found that the over-expression of IPS-1 promoted the induction of the pro-apoptotic gene *TRAIL,* which has been reported to have an important role in the induction of apoptosis in various types of cancers. The over-expression of IPS-1 downregulated the anti-apoptotic genes such as *BCL2*, *BIRC3* and *PRKCE,* known to promote survival in different types of cancer. In contrast, shIPS-1 knockdown cancer cells showed opposite results to those produced by IPS-1 over-expression. We also found that IPS-1 induces caspase activation, which has been shown to be induced by *TRAIL* in various cancer and non-cancer cells.

IPS-1 activation induces type I IFN and inflammatory cytokines, which play essential roles in antiviral immunity. We found that a mutation, C79F, in the CARD of IPS-1 abrogated the ability to induce type I IFN but retained modest ability to induce inflammatory cytokines compared to another mutation, C79I, and the WT IPS-1.^33^ Interestingly, the C79F mutant displayed no anticancer activity, similar to the control. However, the C79I mutant displayed anticancer activity comparable to IPS-1. We further validated our observation by performing the WHA after treating the IMR32 cells with a range of concentrations of rhuIFN*β* and found that rhuIFN*β* induces anticancer activity in a dose-dependent manner. Furthermore, we found that the rhuIFN*β* treatment induced the upregulation of *TRAIL*, which suggested the probable mechanism for the anticancer activity of rhuIFN*β*. On other hand, treatment of anti-IFNAR1 blocking antibody in IFNAR1-suficient cells or complementation of IFNAR1 in IFNAR1-deficient cells resulted in decreased and increase in cytotoxicity, respectively after polyIC transfection. Taken together, these findings suggest that IPS-1 and C79I are critical for the upregulation of type I IFN and induce the upregulation of *TRAIL,* which is essential for the anticancer activity.

Type I IFN have been shown to be dispensable for RLR-induced apoptosis, although the cancer-specific mechanism was not clear.^24^ It has been reported that MDAMB-231 cells lack *IFNAR*1.^11, 12^ We found that the ectopic expression of IPS-1 in the MDAMB-231 cells delayed wound healing and reduced the number of colonies, as indicated by the WHA and colony-formation assay, respectively. Additionally, we found that RLR activation induced *TRAIL* and decreased expression of *BCL2, BIRC3 and PRKCE* in the MDAMB-231 cells. Moreover, NDV replication was found to be significantly higher in the MDAMB-231 cells than in the MCF10A cells. HN protein of NDV induces the expression of TRAIL and type I IFNs.^13^ Infection of NDV in IFNAR1-sufficient cells (MCF10A) induces robust amount of type I IFNs *via* three pathway. First: through sensing RNA of the NDV by RLR sensors. Second, through viral HN protein and third *via* autocrine and paracrine *via* IFNAR1 signaling pathway. Collectively, all these pathways results in sever reduction of the NDV in cells. In contrast, NDV infection in IFNAR1-lacking cells (MDAMB-231) the production of IFN*β* is severely reduced due to absence IFNAR1 signaling pathway *via* IFNAR1 which promotes NDV replication leading to more HN protein and therefore induces strong induction of TRAIL but very low induction of type I IFNs, which further promoted the death of the cancer cells. Collectively, our results suggest that IPS-1 is required for the anticancer activity through the induction of *TRAIL* in a type I IFN-independent manner. ^34^

Study has shown that RLR-dependent IRF3 is critical for inducing apoptosis.^35^ Therefore, we tested the roles of IRF3 and IRF7 in the anticancer activity. The MDAMB-231 cells in which IRF3 and IRF7 were knocked down showed an elevated resistance to apoptosis and reduced *TRAIL* expression after polyIC transfection or NDV infection. IRF3-knockdown cells also showed an upregulation of *BCL2* and *BIRC3,* whereas the IRF7-knockdown cells did not show any change in the expression of these genes, suggesting that IRF7 might not be involved in the regulation of anti-apoptotic genes. However, IRF7 is critical for the RLR-dependent anticancer activity. Previous study suggests that the IRF family transcription factors activation is trans-dependent, which may form a complex for activation of some target promoters.^36, 37^ However, both IRFs might be important for induction of TRAIL promoter. IRF3 might not be directly involved in the downregulation of BCL2 and BIRC3. We tested the induction of various transcription factors involved in the regulation of BCL2 such as p53, SETB2, DDIT3, and found that IRF3 over-expression leads to the upregulation of *p53.* Previous reports suggest that p53, which is induced by type I IFN through IRF3,^23, 38^ directly binds to promoter of BCL2 and downregulates and similar mechanism could be possible for BIRC3 and PRKCE.^15, 16, 17^

**Figure 7:**
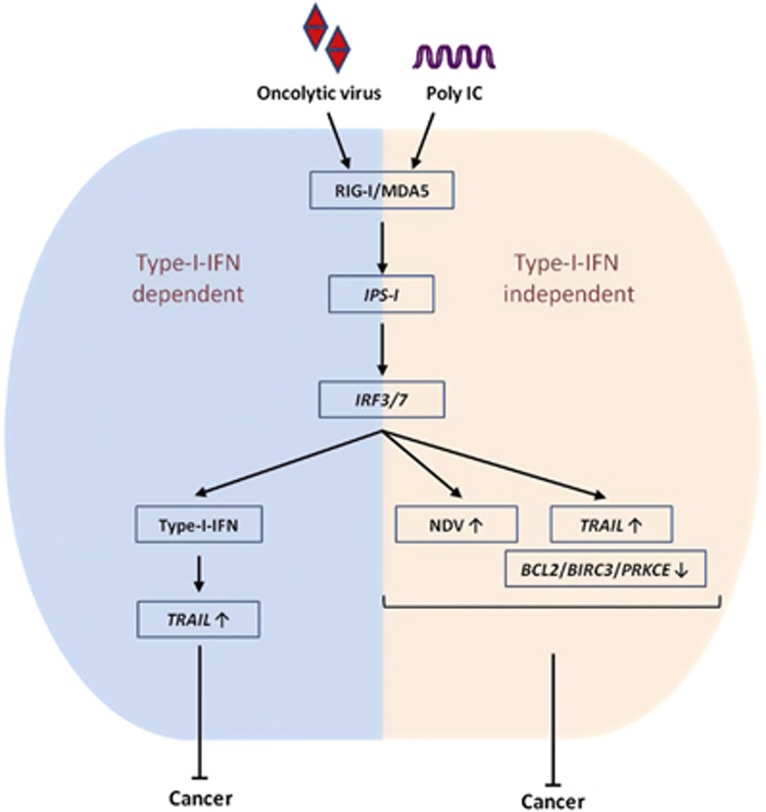
NDV infection and polyIC transfection induce type I IFN-dependent and -independent anticancer activity *via* the IPS-1, IRF3 and IRF7 axis. The mechanism of the type I IFN relies on high NDV replication, upregulation of *TRAIL* or downregulation of the *BCL2*, *BIRC3* and *PRKCE* genes

In conclusion, our findings demonstrated that IPS-1 is critical for anticancer activity, and it is both type I IFN-dependent and IFN-independent. Furthermore, IRF3 and IRF7 positively induce the pro-apoptotic gene *TRAIL* and negatively regulate the anti-apoptotic genes *BCL2*, *BIRC3* and *PRKCE* (Figure 7). Our study also provides the molecular basis of PolyIC- and NDV-mediated cancer therapy, which may be useful in the development of specific therapeutic agent for various cancers.

## Materials and Methods

### Cells, transfection and reagents

MCF10A cells were maintained in 1:1 ratio of Dulbecco's minimum essential medium (DMEM)/F12 supplemented with 10% fetal bovine serum (FBS), 1 × penicillin–streptomycin, 20 ng/ml of epidermal growth factor, 10 *μ*g/ml of Insulin and 0.5 mg/ml of hydrocortisone. MDAMB-231, IMR32 and HEK293T cells were maintained in DMEM supplemented with 10% FBS and 1 × of penicillin–streptomycin. DNA transfection for MDAMB-231, IMR32 and HEK293T was carried out by lipofectamine, as per manufacturer's instructions (Invitrogen, Carlsbad, CA, USA). DMEM, DMEM/F12, FBS, penicillin–streptomycin were purchased from Life Technologies, Carlsbad, CA, USA. 17AAG, epidermal growth factor, insulin and hydrocortisone were purchased from Sigma, St. Louis, MO, USA and MTT from Merck, Kenilworth, NJ, USA.

### Cloning and site-directed mutagenesis

The full length IPS-1 and was cloned into mammalian expression vector pFLAG-CMV2.^1^ pBABE-IFNAR1 plasmids were generously gifted from Prof Akinori Takaoka. IRF3/5D was kind a gift from Dr Toru Kubota. We used PCR mutagenesis standard protocol to create IPS-1 natural variants R43W, R64Q, R65W, G67S, C79I and C79F. All mutants were sequenced, and the clones were verified for the mutation.

### Generation of shRNA-mediated stable knockdown of IPS-1, IRF3 and IRF7 cell lines

shRNA clones, specifically targeting human *IPS-1, IRF3, IRF7* genes and scrambled shRNA were obtained from Mission shRNA, Sigma. MDAMB-231 cells (5 × 10^5^) were electroporated with 30 *μ*g of shRNA plasmids under 250 V and 950* μ*F condition using Gene Pulser, X-cell, Bio-Rad Laboratories (Hercules, CA, USA). The transfectant selected with 0.7 *μ*g/ml puromycin antibiotics for two weeks. The efficacy of each stable shRNA clone to downregulate endogenous expression of IPS-1, IRF3 or IRF7 in respective clones were measured by either semi-quantitative PCR, immunoblot or Immunofluorescence microscopy.

### Immunoblot, co-immunoprecipitation and antibody

Immunoblot and co-immunoprecipitation were performed as described previously.^1^ The antibody against FLAG (A8592) and GAPDH (G9545) were purchased from Sigma. Anti-IPS-1 (3993), anti-PARP-1 (9532), anti-caspase-3 (9662) and anti-caspase-9 (9502) antibody were purchased from Cell Signaling Technology, Beverly, MA, USA. Anti-myc (R951-25) antibody was purchased from Life Technologies. Anti-IFNAR2 monoclonal antibody, clone MMHAR-2 was purchased from PBL assay science, Piscataway, NJ, USA.

### Real time PCR

Total RNA was isolated with Trizol reagent (Invitrogen) and reverse transcribed by using iScriptcDNA synthesis kit (Bio-Rad Laboratories) according to the manufacturer's instructions. PCR was performed with the primers, 28 S (5′-CCGTGTGCAGCCTATCAAG-3′, 5′-TTCTCGCTCTGACTCCAAAAG-3′), Pan-IFN-*α* (5′-CACACAGGCTTCCAGGCATTC-3′, 5′-TCTTCAGCACAAAGGACTCATCTG-3′), IFN*β* (5′-CCTGTGGCAATTGAATGGG-3′, 5′-AACAATAGTCTCATTCCAGCC-3′), IP-10 (5′-TGGCATTCAAGGAGTACCTCTC-3′, 5′-TGATCTCAACACGTGGACAAA-3′), IL-6 (5′-TACATCCTCGACGGCATCTC-3′, 5′-CCAGGCAAGTCTCCTCATTG-3′), TRAIL (5′-AGCAATGCCACTTTTGGAGT-3′, 5′-TTCACAGTGCTCCTGCAGTC-3′),IFNAR1 (5′-CTCCTGTTCCACCTCAGGAT-3′, 5′-ATGGGTGTTGTCCGCAG-3′), IRF3 (5′-ATGCCCTCTGGTTCTGTGTG-3′, 5′-GCTGTTGGAAATGTGCAGGT-3′), IRF7 (5′-GTACGGGTGGGCAGTAGAGA-3′, 5′-GGCCCTTGTACATGATGGTC-3′), TLR3 (5′-TGTGCCAGAAACTTCCCATG-3′, 5′-TGACAAGCCATTATGAGACAGATC-3′) and TP53 (5′-GCCATCTACAAGCAGTCACAG-3′, 5′- TCATCCAAATACTCCACACGC-3′).

### Luciferase reporter assay

HEK293T cells (5 × 10^4^) were seeded into 24-well plate and transiently transfected with 50 ng transfection control pRL-TK plasmid and 100 ng luciferase reporter plasmid along with 500 ng various expression plasmids or empty plasmid. The cells were lysed at 24–36 h post transfection and luciferase activity in total cell lysate was measured by using Glomax (Promega, Madison, WI, USA).

### Confocal microscopy

Cells were stained as described previously^39^ and confocal fluorescent images were obtained by LSM 780 confocal microscope (Carl Zeiss, Jena, Germany).

### Fluorescence-activated cell sorting (FACS)

Cells were analyzed by staining with FITC-labeled Annexin V and propidium iodide (Becton Dickinson, USA) as per manufacturer's instructions and stained cells were analyzed using a FACS Aria III (Becton Dickinson) and data were analyzed by using FlowJo software (FlowJo, Ashland, OR, USA).

### Cell viability assay

MTT assay were performed as per described previously^19^ to determine cell viability. Values shown are the mean S.D. of at least eight measurements.

### NDV virus infection

MDAMB-231 cells were infected with NDV/lasota at different MOI, or they were mock-infected with phosphate-buffered saline, at 37 °C for 1 h in serum-free DMEM. The cells were washed with phosphate-buffered saline and incubated at 37 °C in reduced serum (1% FBS) media. Cells were harvested after 24 h post infection for further analysis.

### Enzyme-linked immunosorbent assay (ELISA)

IMR32 and MDAMB-231 cells were transiently transfected with IPS-1 and culture supernatants were harvested after 36–40 h and analyzed for production of IP-10 by ELISA according to manufacturer's instruction (Becton Dickinson).

### Wound healing assay

The cells were seeded into six-well plate and it was transfected at 100% confluency with 2 *μ*g plasmid or 4 *μ*g polyIC or stimulated with 25* μ*g polyIC. After 6 h of transfection or stimulation, the wounds were scratched using 200-*μ*l tip and three random images of wound were taken after two washes with media using × 10 objective lens. The images were taken at every 24-h time intervals till the wound in control is completely healed.

### Soft agar assay

Cells were suspended at a concentration of 1 × 10^4^ cells/ml in agarose over a layer of agar, as described^40^ and grown for 20 days.

### Clonogenic assay

MDAMB-231 (1000 cells/well) were seeded in 6-well plate and incubated for 5 days. The cells were transfected with 2 *μ*g polyIC and incubated again 5 days. After completion of experiment, cells were washed two times with phosphate-buffered saline and fixed in 3:1 ratio of methanol:acetic acid for 5 min. The fixed cells were stained with 0.05% crystal violet in methanol for 15 min. The stained cells were washed and scanned to determine the number of colonies.

### Invasion assay

The MDAMB-231 cells were transfected or treated as per experiment along with appropriate control thereafter 1 × 10^4^ cells were added to the upper side of transwell over Matrigel layer and incubated for 24 h in tissue culture incubator. The non-migrated cells in upper layer of Matrigel were removed and migrated cells to the transwell filter were fixed in 4% PFA, stained with 0.05% crystal violet and 10 random fields were counted under light microscopy.

### Statistical analysis

All experiments were carried out along with the appropriate controls, indicated as untreated cells (Ctrl) or transfected with the transfection reagent alone (Mock). Experiments were performed in duplicates or triplicates for at least three times independently. GraphPad Prism 5.0 (GraphPad Software, La Jolla, CA, USA) was used for statistical analysis. The differences between two groups were compared by using an unpaired two-tailed Student's *t*-test, while differences between three groups or more were compared by using analysis of variance with Newman–Keuls test. Differences were considered statistically significant with a *P*-value <0.05. ***P<0.001, **P<0.01, *P<0.05, ns=not significant (unpaired two-tailed Student's *t*-test).

## Abbreviations

IPS-1: interferon-*β* promoter stimulator-1
RLRs: RIG-I-like receptors
polyIC: polyinosinic:polycytidylic acid
NDV: Newcastle disease virus
TRAIL: tumor necrosis factor-related apoptosis-inducing ligand
BCL2: B-cell lymphoma 2
BIRC3: baculoviral IAP repeat-containing protein 3
PRKCE: protein kinase C epsilon
