# Supplementary figures and images for: IPS-1 differentially induces TRAIL, BCL2, BIRC3 and PRKCE in type I interferons-dependent and -independent anticancer activity
Source: Cell Death Dis. 2015 May 7;6(5):e1758–. doi: 10.1038/cddis.2015.122 (PMC4669701; doi:10.1038/cddis.2015.122)

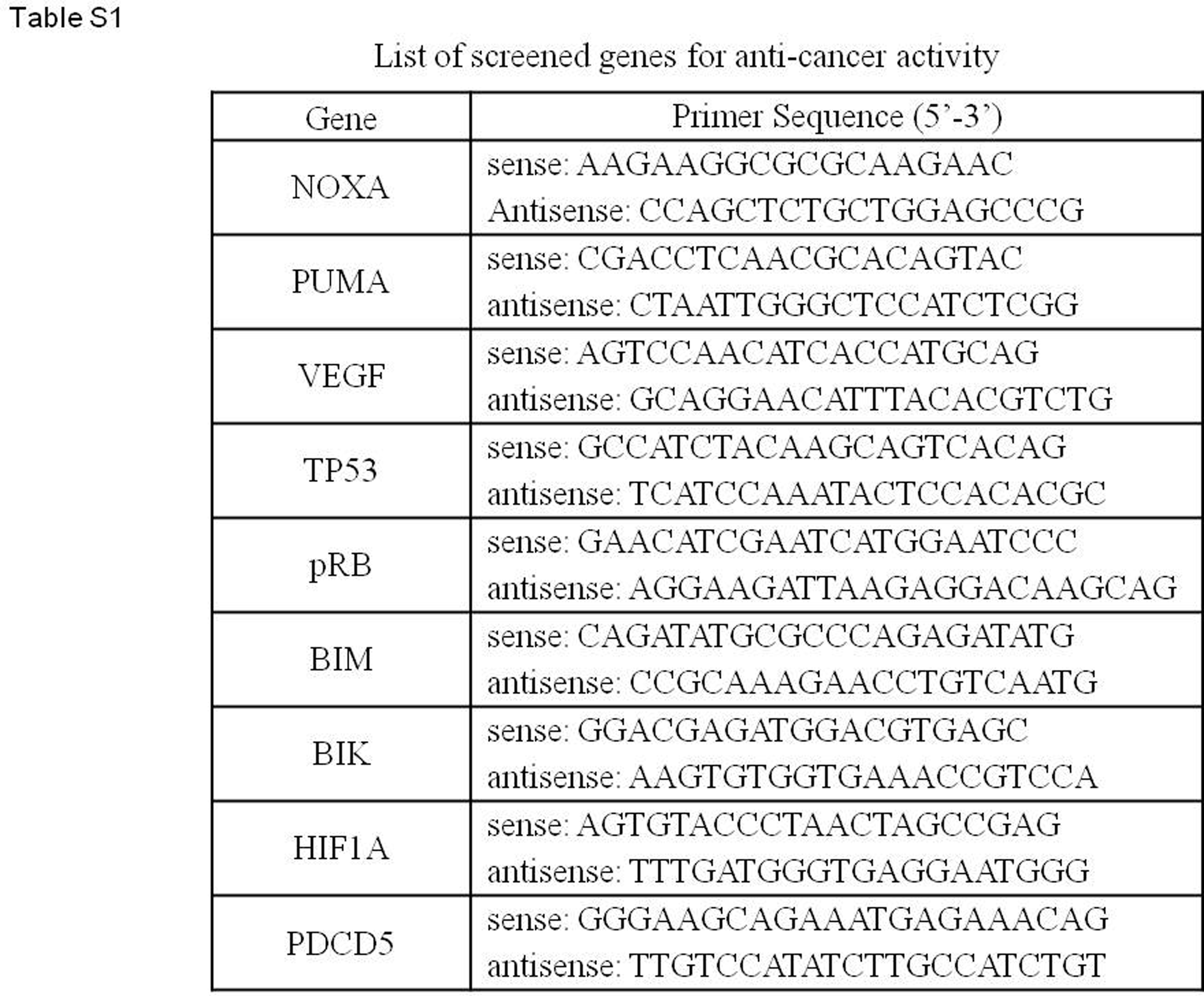

Supplement: Supplementary Table S1 [file cddis2015122x1.tif]

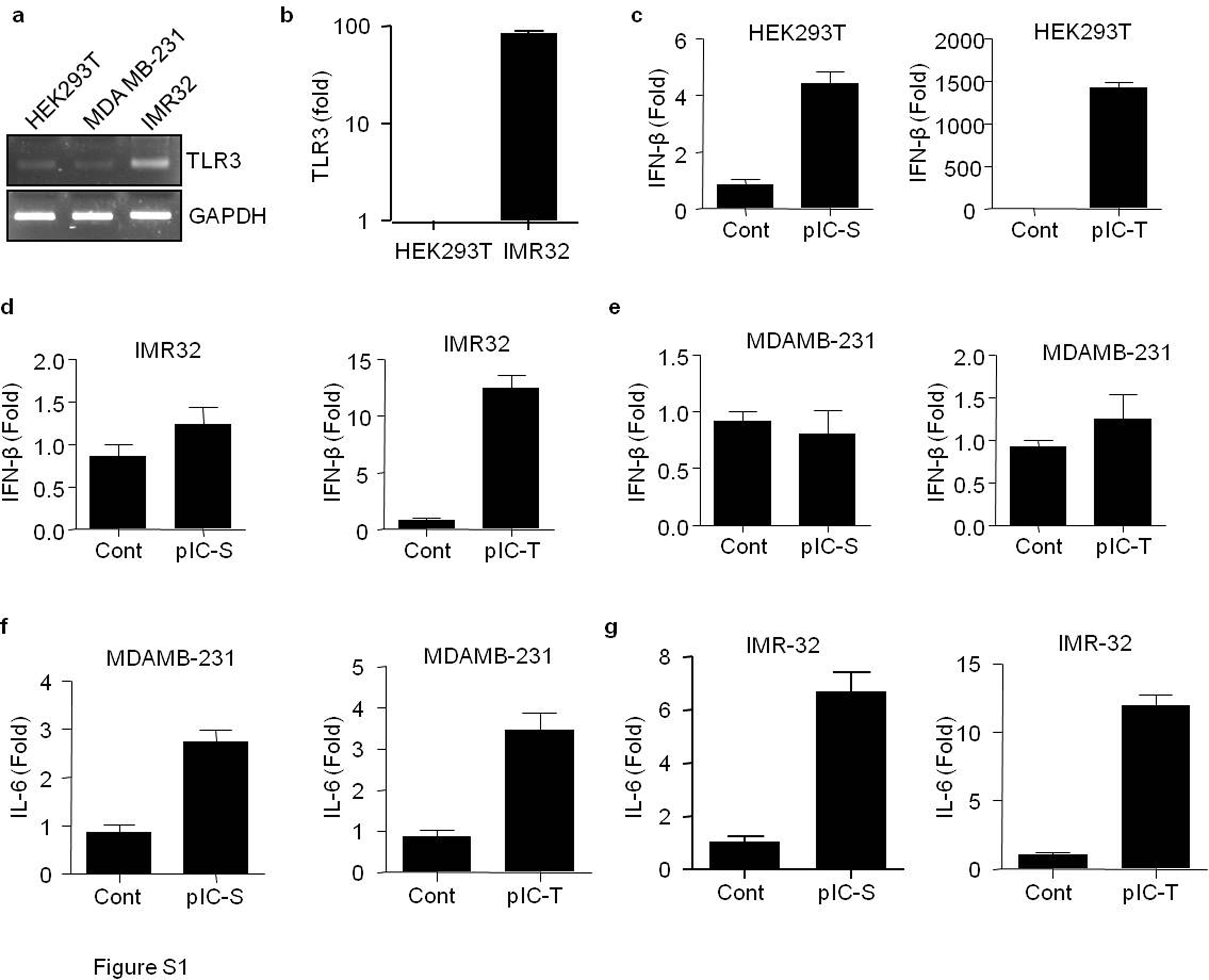

Supplement: Supplementary Figure S1 [file cddis2015122x2.tif]

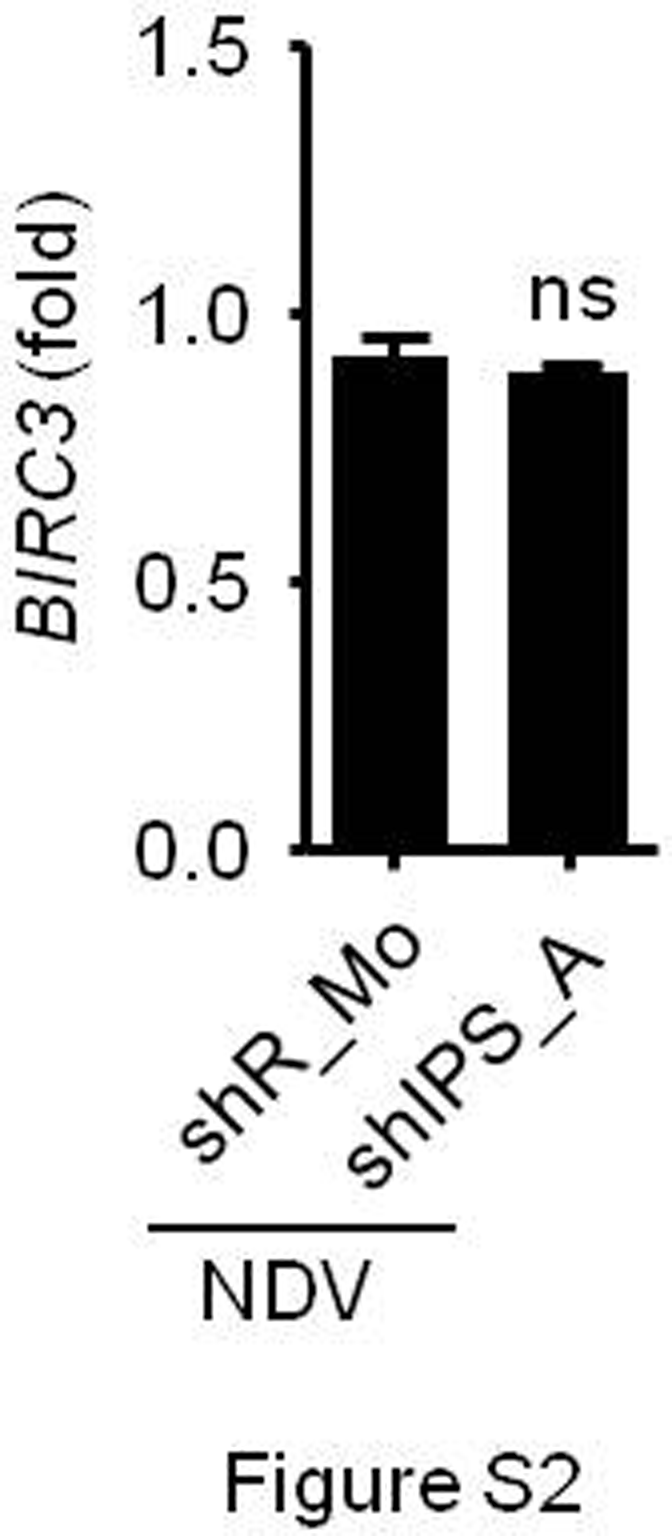

Supplement: Supplementary Figure S2 [file cddis2015122x3.tif]

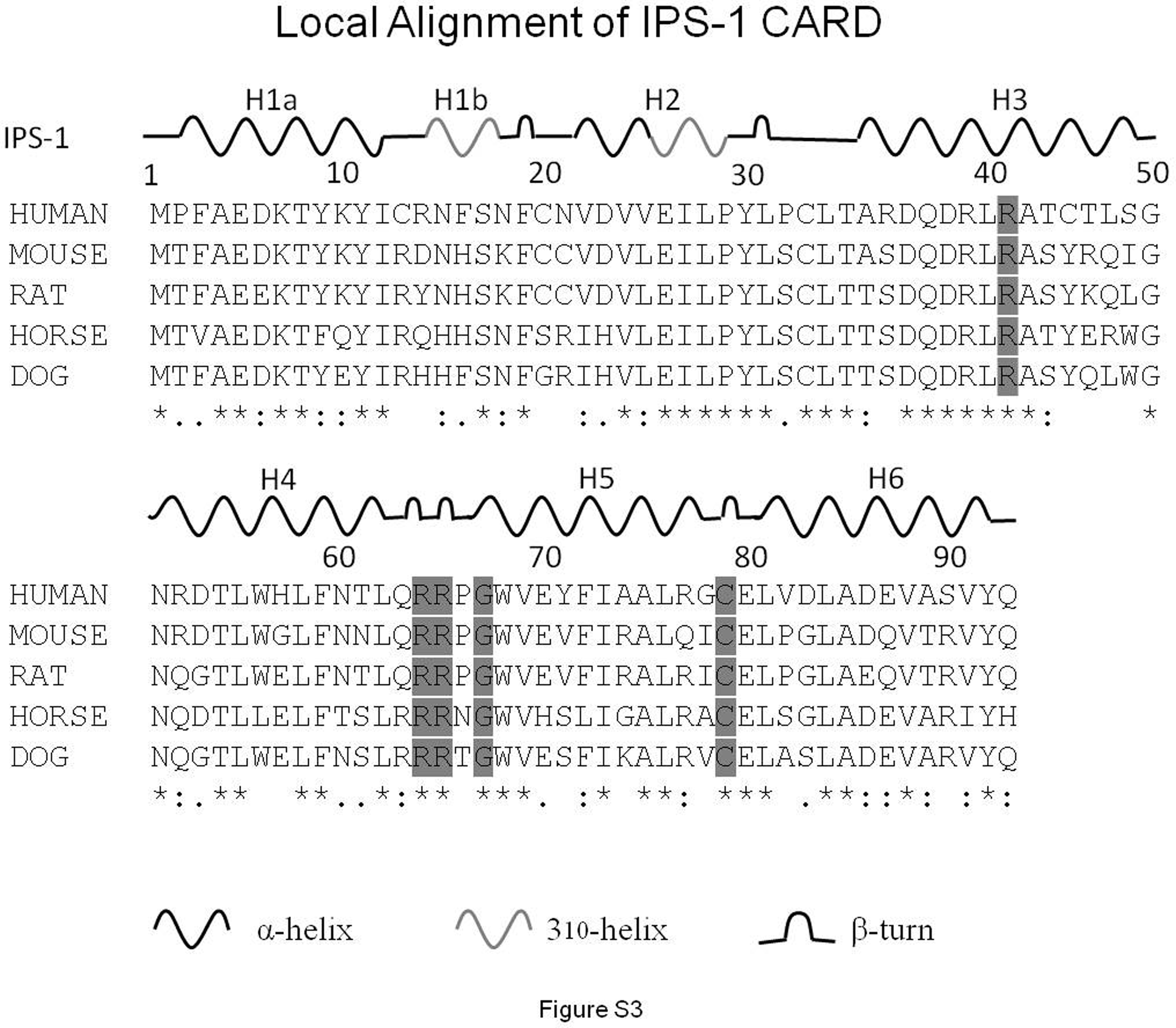

Supplement: Supplementary Figure S3 [file cddis2015122x4.tif]

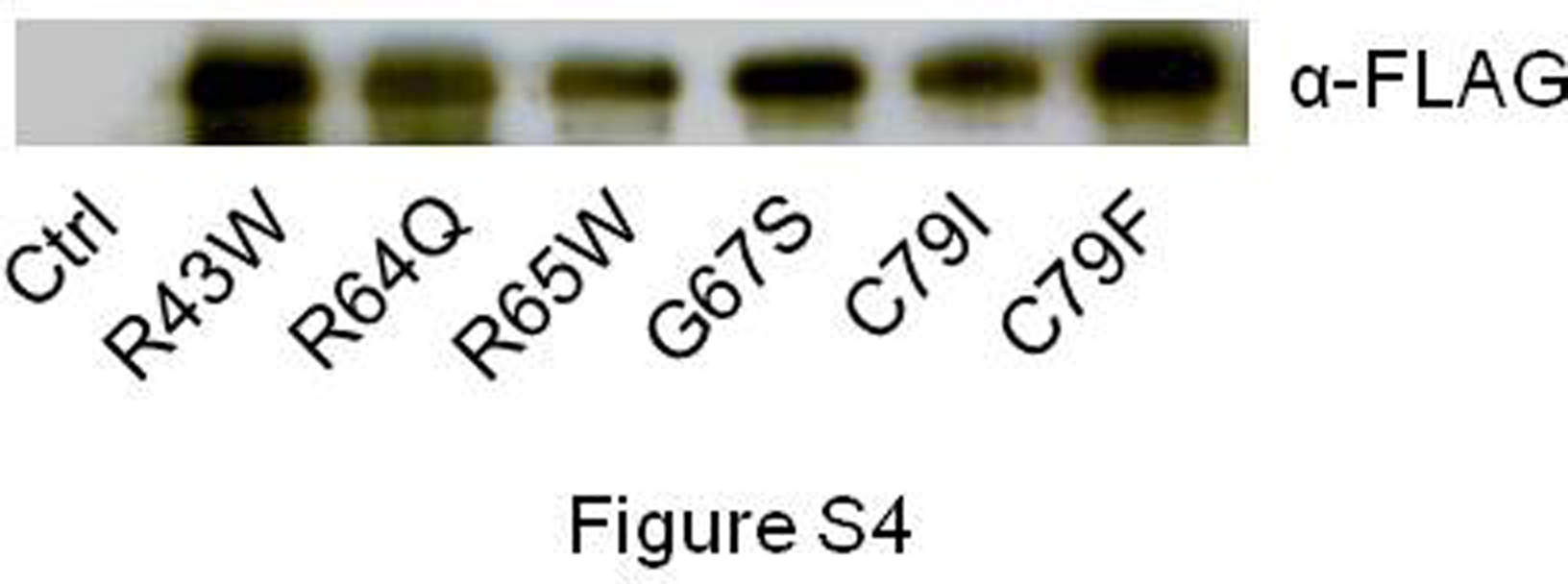

Supplement: Supplementary Figure S4 [file cddis2015122x5.tif]

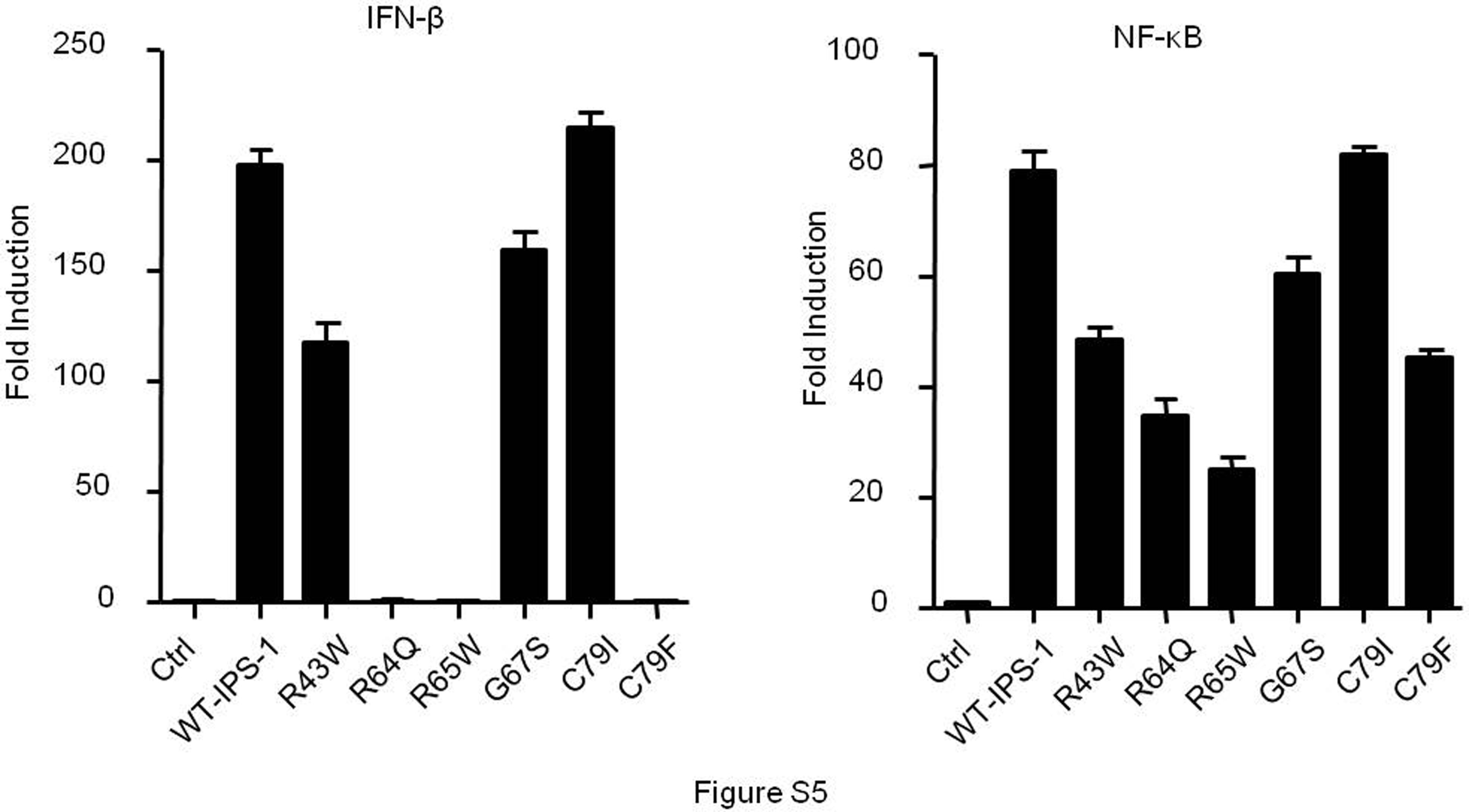

Supplement: Supplementary Figure S5 [file cddis2015122x6.tif]

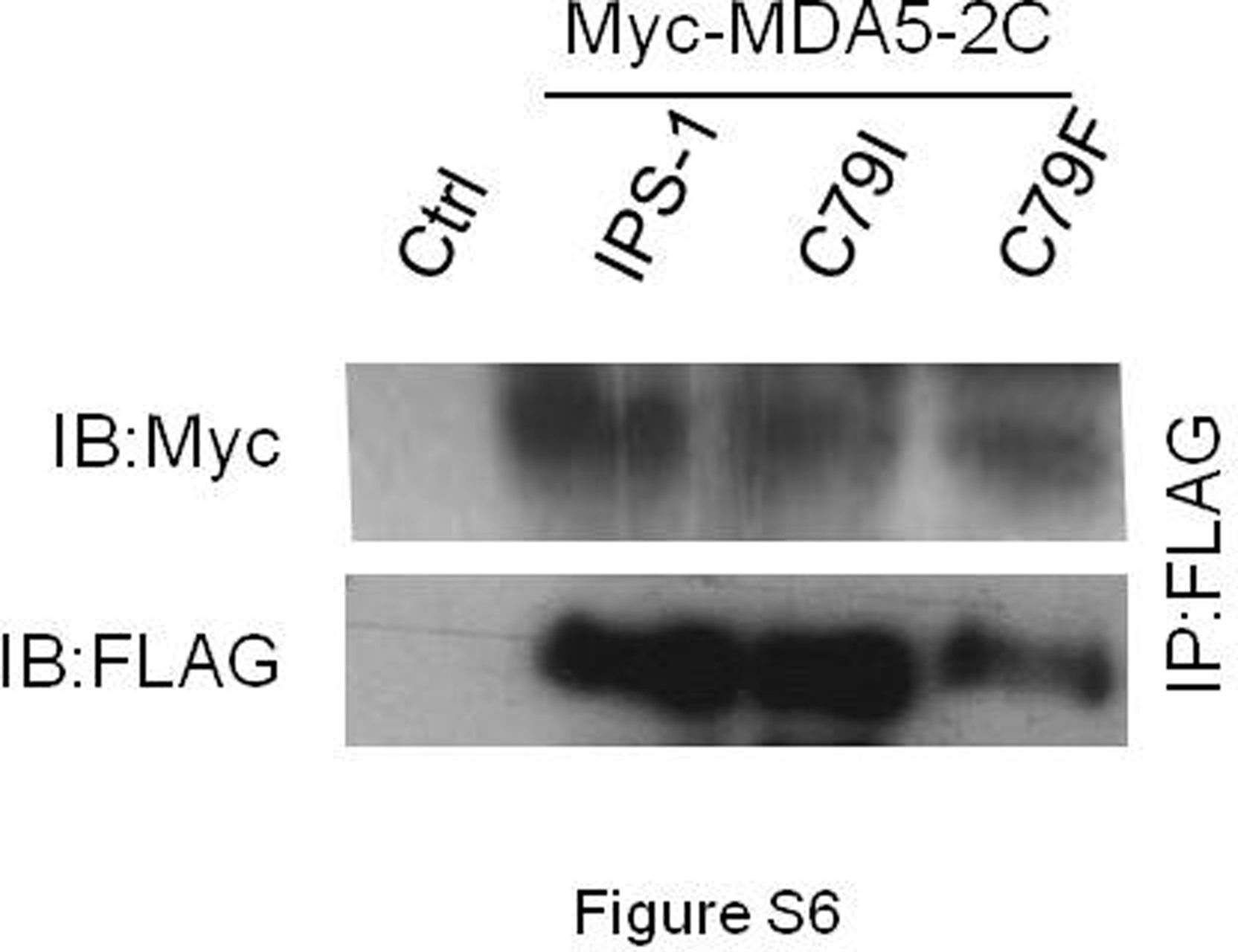

Supplement: Supplementary Figure S6 [file cddis2015122x7.tif]

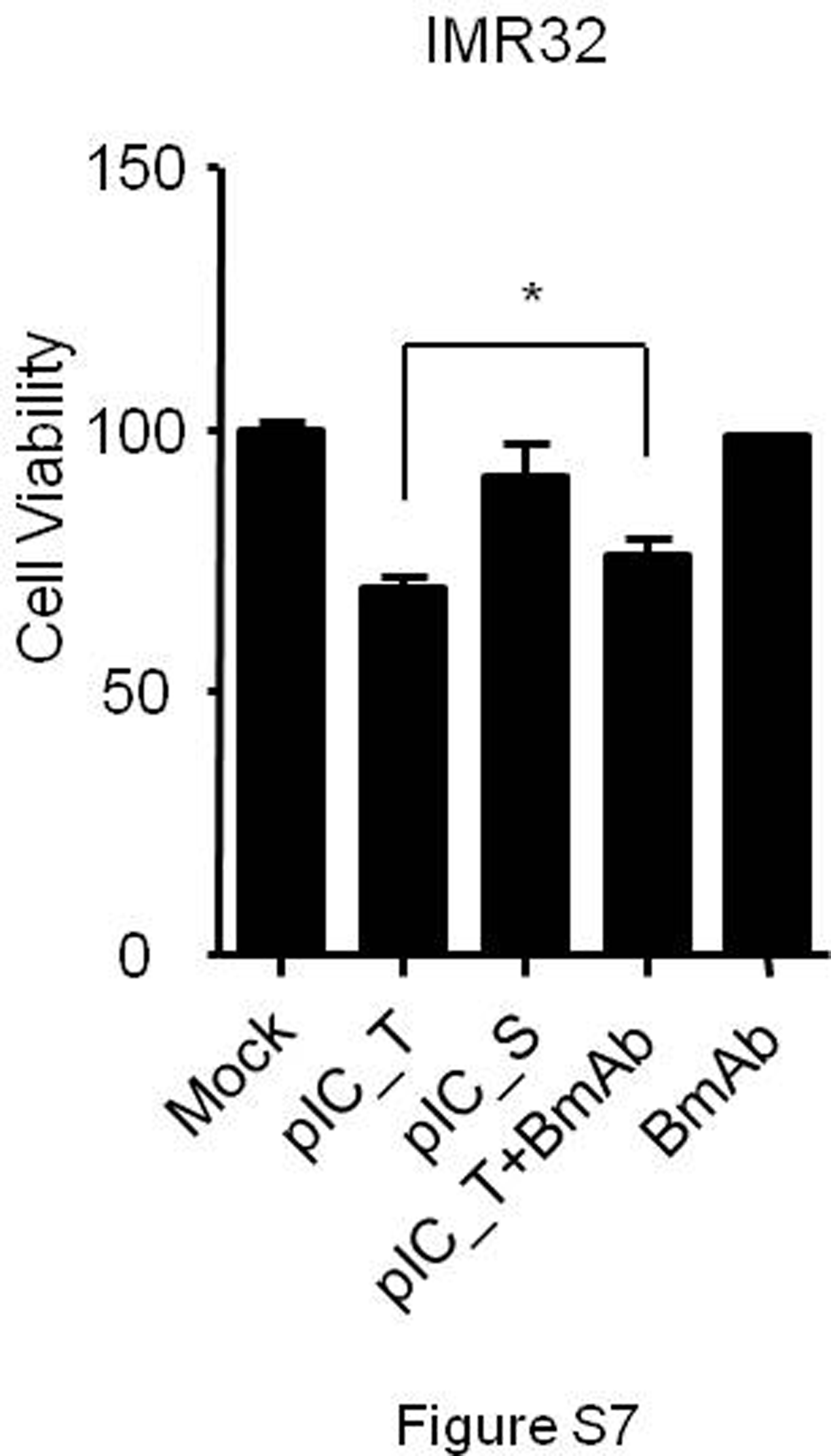

Supplement: Supplementary Figure S7 [file cddis2015122x8.tif]

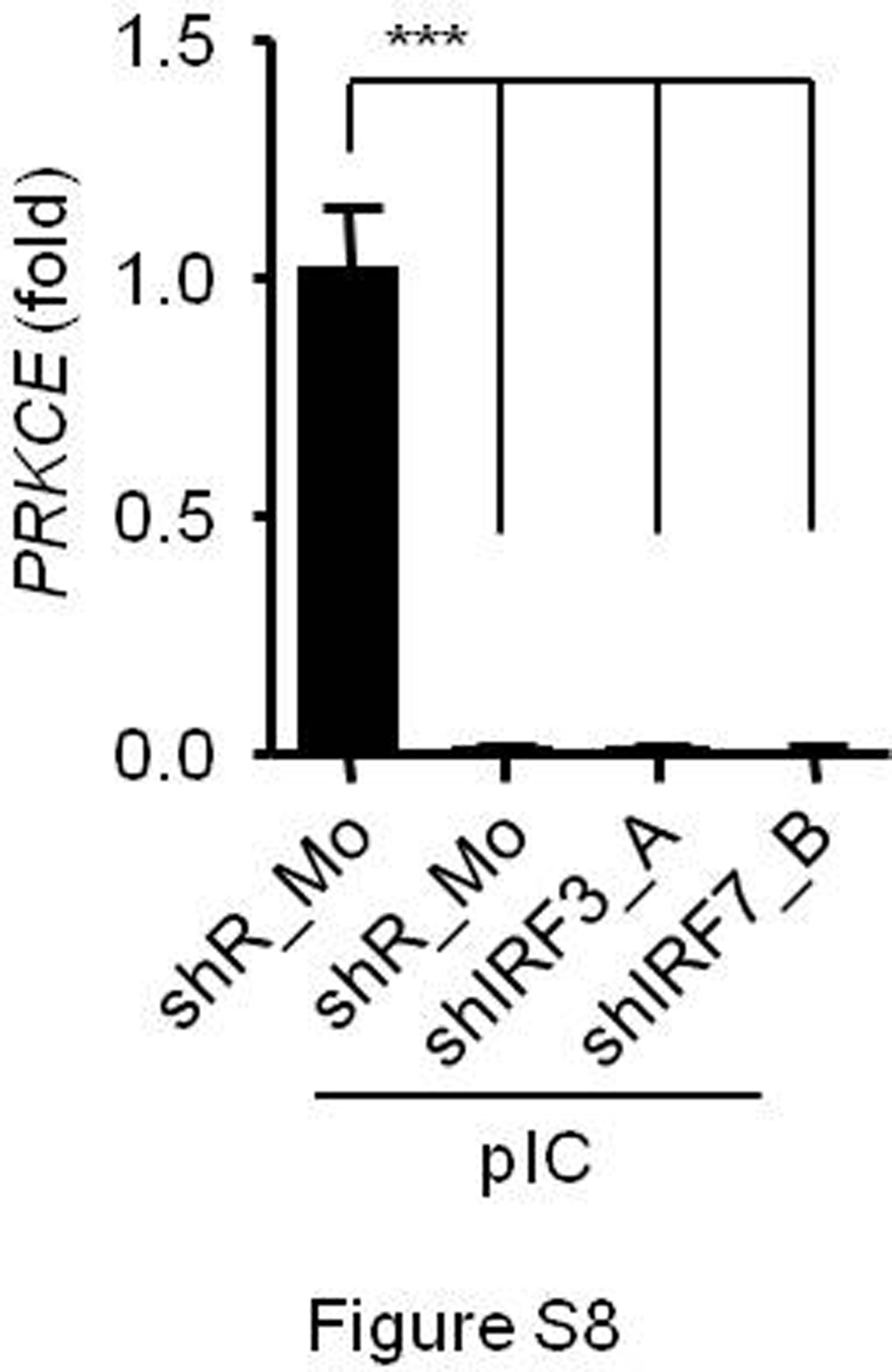

Supplement: Supplementary Figure S8 [file cddis2015122x9.tif]

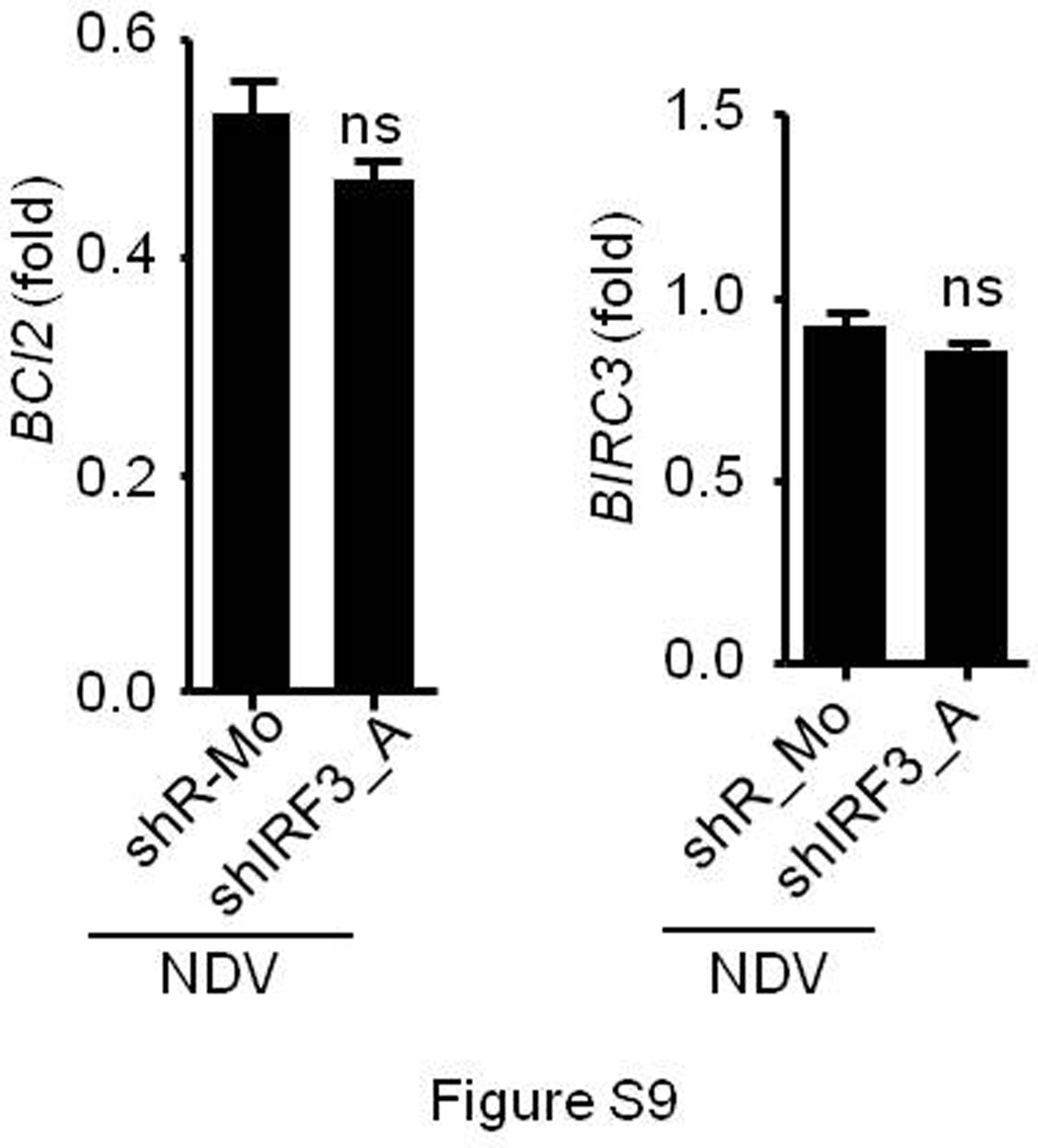

Supplement: Supplementary Figure S9 [file cddis2015122x10.tif]
