# Supplementary material for: IPS-1 differentially induces TRAIL, BCL2, BIRC3 and PRKCE in type I interferons-dependent and -independent anticancer activity
Source: Cell Death Dis. 2015 May 7;6(5):e1758–. doi: 10.1038/cddis.2015.122 (PMC4669701; doi:10.1038/cddis.2015.122)
Supplement: Supplementary Figure Legends [file cddis2015122x11.doc]

**Figure S1:** Figure 1. Cells were lysed and the mRNA level of TLR3 was quantified by semi-quantative PCR (a) or by qRT-PCR (b). (c, d, e, f, g) Indicated cell lines were transfected or stimulated with polyIC, after 6h, mRNA levels of IFN-β or IL-6 were quantified using qRT-PCR.

**Figure S2:** shIPS_A cells were infected with 5MOI of NDV, after 24h, cells were lysed and mRNA levels of *BIRC3* were quantified using qRT-PCR.

**Figure S3:** Alignment of IPS-1 CARD protein sequences from the indicated organisms. Conservation as indicated by ClustalW software is shown at the bottom using * (asterisk) to indicate fully conserved residues, : (colon) to indicate conservation between groups of strongly similar properties and . (period) to indicate conservation between groups of weakly similar properties. The IPS-1 CARD protein secondary structure is shown in the upper panel.

**Figure S4:** HEK293T cells were transfected with the indicated IPS-1 mutants or left untransfected (control). After 24h, the cells were lysed, and the FLAG-IPS-1 protein levels were quantified using immunoblot with an anti-FLAG antibody.

**Figure S5:** HEK293T cells were seeded into a 24-well plate and transiently transfected with the indicated IPS-1 mutant expression plasmids (500 ng/well) along with 100 ng of a reporter construct containing IFNβ (left) or NF-κB (right) with the transfection control pRL-TK plasmid. After 24h, the cells were lysed, and the promoter activities were analyzed using the luciferase assay. All experiments were performed in duplicate or triplicate and similar results were obtained from four independent experiments.

**Figure S6**  HEK293T cells were transiently transfected with the combination of FLAG-tegged IPS-1, C79I or C79F along with Myc-tagged MDA5_2CARD (MDA5_2C) plasmid, after 36h, cell lysates were immunoprecipitated (IP) with anti-FLAG antibody and immunoblot (IB) with anti-Myc and anti-FLAG antibody.

Figure S7 IMR32 cells were treated with anti-IFNAR2 blocking antibody before polyIC transfection and stimulation, after 30h, cell viability assay was performed.

**Figure S8** shIRF3-A, shIRF7_B and shR_Mo cells were transfected with pIC, after 6h, mRNA levels of *PRKCE* gene was quantified using qRTPCR.

**Figure S9** shIRF3-A and shR_Mo cells were infected with NDV, after 6h, mRNA levels of indicated genes were quantified using qRTPCR.
